# Investigating the Mechanical Property and Enhanced Mechanism of Modified Pisha Sandstone Geopolymer via Ion Exchange Solidification

**DOI:** 10.3390/gels8050300

**Published:** 2022-05-13

**Authors:** Changming Li, Lisha Song, Yali Cao, Shunbo Zhao, Hui Liu, Chen Yang, Haifeng Cheng, Dongyang Jia

**Affiliations:** 1 School of Civil Engineering and Transportation, North China University of Water Resource and Electric Power, Zhengzhou 450045, China; yhzhanghky@163.com (Y.C.); sbzhao@ncwu.edu.cn (S.Z.); chf6077@163.com (H.C.); dongyangjia2022@163.com (D.J.); 2 Department of Civil Engineering, Henan Technical College of Construction, Zhengzhou 450064, China; songlisha0202@163.com; 3 Engineering and Technical Research Center of Levee Safety and Disease Control, Yellow River Institute of Hydraulic Research, Zhengzhou 450003, China; 4 Collaborative Innovation Center for Efficient Utilization of Water Resources, North China University of Water Resources and Electric Power, Zhengzhou 450046, China; yangchen@ncwu.edu.cn

**Keywords:** Pisha sandstone, ion exchange, solidification and strength development, geopolymer gel

## Abstract

The Yellow River has the highest sediment concentration in the world, and the Yellow River coarse sediment mainly comes from a particular kind of argillaceous sandstone, Pisha sandstone. This paper reports an investigation of the possibility of development of low-cost engineering materials using Pisha sandstone via ion exchange modification. The effect of modifiers with different concentration on the inhibition of volume expansion and the strength enhancement of modified Pisha sandstone were studied via ion exchange solidification. The effects of the concentration of ten types of modifier solutions and curing age were considered. The hydration of the mineral components, particle surface potential and reaction products were studied, respectively, by XRD, zeta potential, TG/DTG and SEM. Expansion volume and shear strength tests were conducted to assess the volume stability and mechanical property of modified Pisha sandstone. It showed that the expansion of Pisha sandstone was controlled and that the volume stability and shear strength were improved via ion exchange modification. The results of XRD, TG/DTG and SEM showed that the spacing of the crystal layers of the Pisha sandstone clay mineral and the mass lost had decreased significantly. When the concentration of the modifier was 0.05 mol/L, the volume reduced by 54.55% maximum and the shear strength reached the peak of 138 kPa.

## 1. Introduction

The Loess Plateau, the third largest plateau in China, is located in the north-west of China with an area of 640,000 km^2^. It is one of the regions with the most serious soil erosion and the most fragile ecological environment in the world. In the lengthy period of intense erosion, the landforms with thousands of gullies and fragmented terrain gradually formed. The Yellow River, the fifth largest river in the world, flows through the Loess Plateau. A large amount of sediment was carried into the Yellow River by flooding. The Yellow River has the highest sediment content and is famous as an overhanging river. According to Wang Hao’s research [1], there was about 1.6 billion tons of sediment taken into the Yellow River from the Loess Plateau by flood every year in the middle of the 20th century. Although, many measures had been taken to reduce the amount of sediment entering the Yellow River, on average there were 750 million tons of sediment taken annually into the Yellow River between the 1980s and 2000s, and the annual average amount of sediment entering the Yellow River was still as high as 240 million tons in the last twenty years. It was reported that the sediment deposited on the Yellow River bed was mainly coarse sediment with a particle size greater than 0.05 mm [2]. This coarse sediment deposited on the Yellow River bed was found to come from a certain area in the Loess Plateau. Bi and Ran [3,4] further noted that the coarse sediment producing area was located on the border of the Inner Mongolia, Shanxi and Shaanxi provinces, covering an area of 20,000 km^2^ [3,4,5]. The reason the coarse sediment producing area has the most serious soil erosion in the Loess Plateau is that there is a special kind of argillaceous sandstone, Pisha sandstone, distributed throughout this area.

Pisha sandstone (PS) is a particular kind of argillaceous sandstone which formed during the Tertiary period [6]. Pisha sandstone can be divided into white, incanus, daffodil yellow, red and green in color, and it is composed of the amorphous clay minerals (montmorillonite, illite and kaolinite, etc.) and crystalline minerals (feldspar, quartz and calcium, etc.). PS has a weak bonded mechanism and its petrographic structure is unsatisfactory, thus, it is a very hard rock when it is dry. However, it collapses into sand soon after being immersed in water due to the poor bonded mechanism and the expansion of clay minerals [7,8]. Hence, the ecology of the PS area is very poor and the soil erosion is very severe. The PS area has been the concentrated source area of coarse sediment in the middle reaches of the Yellow River. Moreover, the PS area is also a typical multi dynamic compound erosion area in the Loess Plateau of China. It includes many erosion types such as hydraulic erosion, wind erosion, freeze–thaw erosion and gravity erosion. Because water erosion, wind erosion and freeze–thaw erosion occur alternately, the PS area has severe soil erosion and extremely poor ecological environment. The soil erosion modulus reaches 30,000 to 40,000 t/(km^2^∙a) [8]. Substantial amounts of PS are being broken into sediment by floods and deposited on the Yellow River course so that the altitude of the riverbed is raised every year. Although the PS area accounts for only 2.6% of the Loess Plateau, it contributes 30% of the coarse sediment of the Yellow River [9,10]. Therefore, the PS area has been described by researchers as a life exclusion zone which is suffering from an “Earth environment cancer”.

The soil erosion and ecological degradation in the PS area have had a seriously poor influence on the development of the local economy. In order to control the soil erosion and improve the ecological environment of the PS area, a number of researchers investigated the work efficiency of the treatment measures aimed at controlling the soil erosion and repairing the eco-environment, such as sea-buckthorn flexible dams, biological engineering measures, check dam and so on. It showed that the check dam had the highest work efficiency among all governance measures [11,12,13,14]. Thus, several check dams were planned by the local government in the PS area. The demand of damming materials for check dams has been significant. However, due to the fragmented terrain and the terrible traffic transport infrastructure, traditional dam materials are scarce, and the cost of traditional building materials such as concrete is very high. Compared with traditional damming material, PS is easier and more widely available all over the PS area. Compared with traditional materials used for the location check dams, PS has a huge economic advantage, is more available and more environmentally friendly. However, most of the existing check dams which were constructed directly with PS were damaged frequently during floods because the PS would expand and collapse in water [15]. In order to solve the problem that PS could not be directly used for damming, many studies have been conducted to reveal the expansion and collapse mechanism of PS. Ye Hao and Shi Yingchun et al. [16] studied the influence of PS’ lithology on corrosion resistance and analyzed the internal causes of PS erosion. Wang Lijiu and Yao Wenyi et al. [17,18,19] conducted a systematic study on the anti-corrosion and growth-promoting governance model in the PS area.

Geopolymer is a green, effective and environmentally friendly material with high strength, corrosion resistance and high temperature resistance [20,21,22,23,24,25,26,27,28]. A few researchers paid attention to developing modified PS materials based on alkaline activated technology [29,30,31]. Nevertheless, the alkaline activated modified PS materials were difficult to break down and would be retained for such a long time that it would affect vegetation recovery and growth. Moreover, it is difficult to produce geopolymer gels by using PS due to its low pozzolanic activity. PS cannot be directly used as dam building material because of its property of collapse in water. Therefore, it would be a beneficial exploration to control the volume stability of PS, improve its mechanical strength and develop it into a kind of naturally degradable engineering geopolymer material.

The purpose of this work is to develop a new kind of naturally degradable modified PS materials via ion exchange solidification to investigate the effect of modifier types and concentration at different curing ages on the volume expansion inhibition and mechanical strength enhanced of modified PS, and to see how they influence the development of volume stability, mechanical strength and reaction products of PS. It is hoped that the processing of modified PS will be well understood and will offer the optimal scheme of manufacture for naturally degradable engineering geopolymer materials by using PS.

## 2. Results and Discussion

### 2.1. Volume Reduction Rate

**Figure 1:**
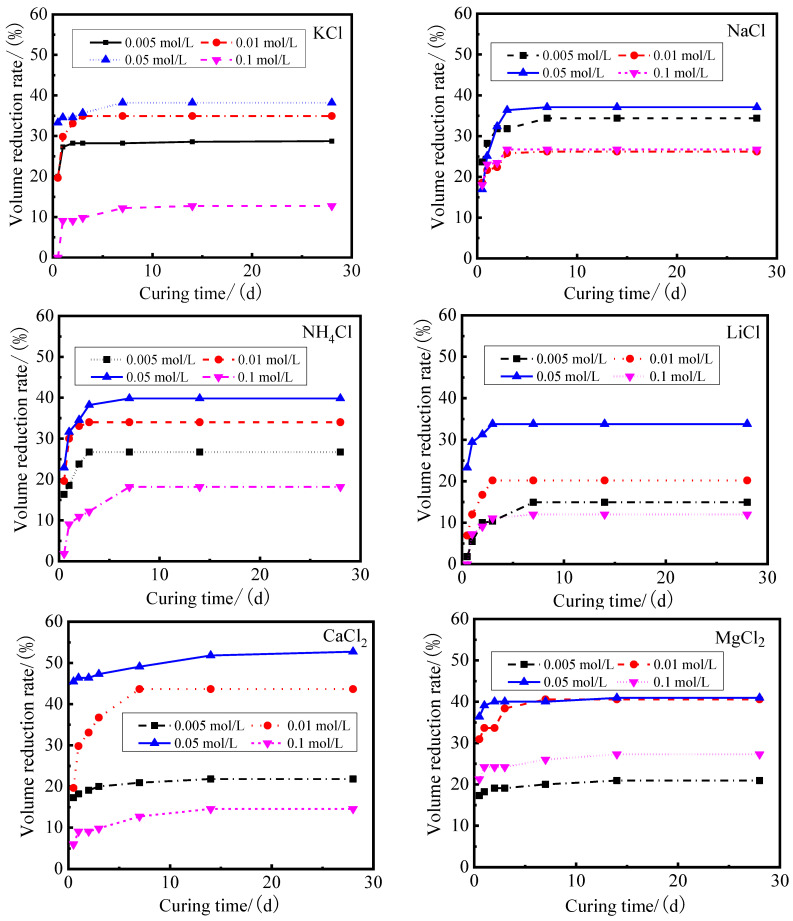

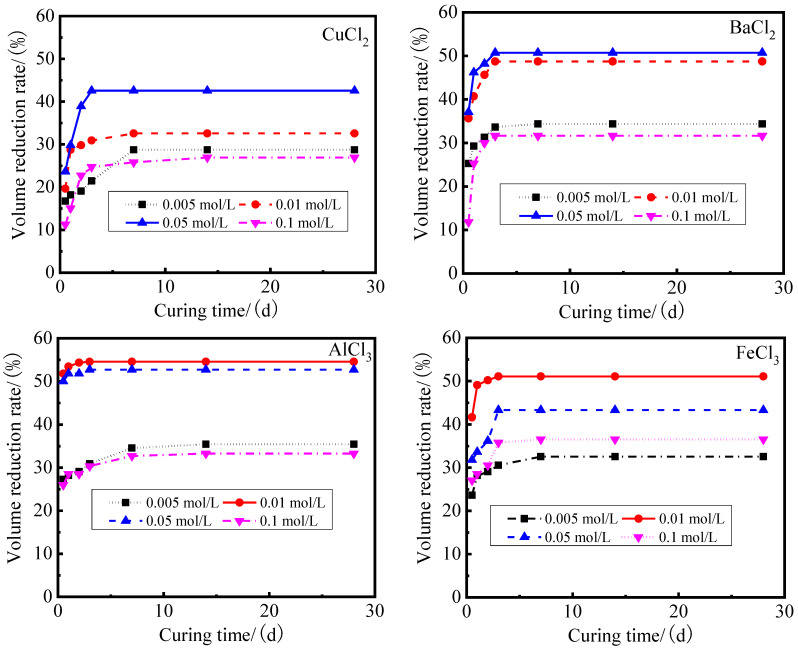
Volume reduction rate of modified PS samples based on different modifiers.

The volume reduction rate (the ratio of the volume reduction of modified sample to the volume of the sample in distilled water) of modified PS samples based on different modifiers is shown in Figure 1. It can be seen that the volume reduction rate of the samples has a negligible change after 7 days. The final volume reduction rate of the samples in distilled water was 0%, and the volume reduction rate of the samples in the modifier solution had significantly increased when compared to the samples in distilled water. The volume reduction rate of modified PS samples changed between 9.09% and 54.55%. For the modified PS samples based on KCl, NaCl, NH_4_Cl and LiCl, the maximum volume reduction rates were 38.18%, 37.09%, 39.82% and 33.82%, respectively. For the samples modified by the divalent modifiers, such as CaCl_2_, MgCl_2_, BaCl_2_ and CuCl_2_, the maximum volume reduction rates were 52.73%, 40.91%, 50.73% and 42.55%, respectively. The maximum volume reduction rates of the samples based on the trivalent chloride salt AlCl_3_ and FeCl_3_ modifiers were 54.55% and 51.09%, respectively.

**Figure 2:**
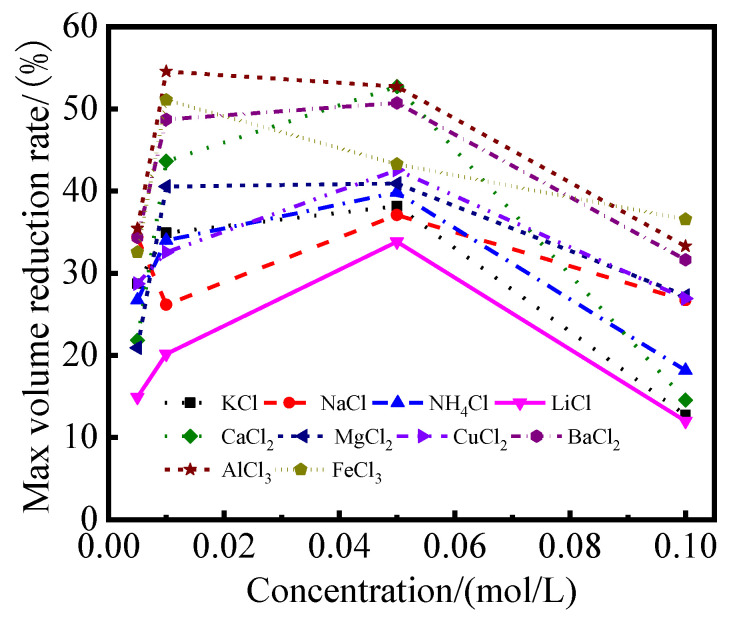
Effect of modifier solution concentration on maximum volume reduction rate of samples based on different modifiers.

Figure 2 depicts the relationship between the maximum volume reduction rate (the ratio of the maximum volume reduction of modified sample to the volume of the sample in distilled water based on different modifiers at a fixed concentration) of modified PS samples and the modifier concentration. It can be seen that the maximum volume reduction rate of modified PS samples increased first and then decreased with the increase of modifier concentration. For monovalent modifiers (e.g., KCl, NaCl, NH_4_Cl and LiCl), the maximum volume reduction rate of samples varied with the concentration of the modifier between 3% and 40%. The volume reduction rate of samples reached the highest value when the concentration of the modifier solution was 0.05 mol/L. For the samples based on divalent modifiers, (e.g., CaCl_2_, MgCl_2_, CuCl_2_, BaCl_2_, AlCl_3_ and FeCl_3_), the maximum volume reduction rate of samples varied between 6% and 48%. The maximum volume reduction rate of all modified PS samples reached the highest value when the concentration of the modifier solution was 0.05 mol/L (except for MgCl_2_). For the samples modified by the trivalent modifiers, such as AlCl_3_ and FeCl_3_, the maximum volume reduction rate varied between 25% and 55%, and the maximum value was obtained when the concentration of the modifier solution was 0.01 mol/L. As shown in Figure 2, the trivalent modifier obtained the best results on the volume stability improvement in all modifiers.

### 2.2. Effect of Ion Type

**Figure 3:**
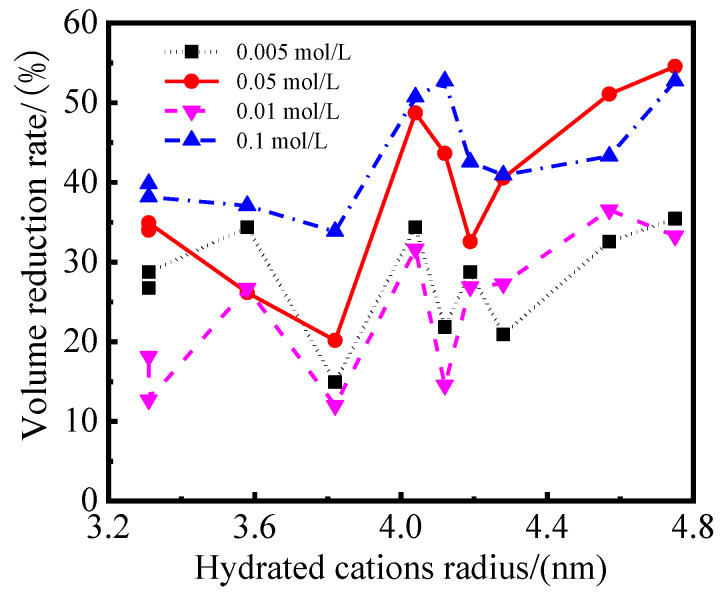
Relationship between hydrated cations radius and volume reduction rate of samples.

Figure 3 presents the relationship between the hydrated cations radius and the volume reduction rate of the modified PS samples. Figure 3 shows that the volume reduction rate of the samples increased with the increase of the hydrated cations radius. Usually, when the radius of the cations crystalsis smaller, the hydration capacity, the thickness of the hydration film and the expansion of the samples is stronger and greater [32,33]. However, the hydration expansion of cations is not only related to the radius of the hydrated cations, but also to the charge density of the cations’ surface.

**Figure 4:**
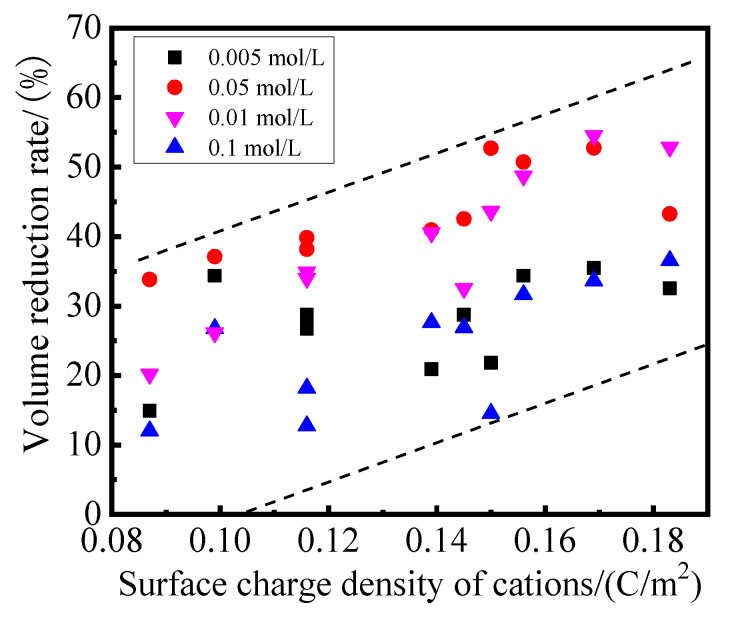
Relationship between surface charge density of cations and volume reduction rate of samples.

The relationship between the surface charge density of hydrated cations (the ratio of the quantity of charge to the surface of hydrated cations [34]) and volume reduction rate of modified PS samples was shown in Figure 4. It can be seen that the volume reduction rate of the samples based on different modifiers increased with the increase of the surface charge density of hydrated cations. The surface charge density of cations had a significant effect on the hydration degree between cations and water molecules: the higher the surface charge density of cations were, the thicker the surface hydration film of cations [34]. On the one hand, the surface charge density of cations significantly affects the electrostatic interaction between cations and the surface of the crystal layer of the sample. In other words, the higher the cation surface charge density was, the stronger the electrostatic interaction between the surface of the crystal layer of the sample was when other conditions remained unchanged. In this way, the adsorption of cations on the surface of the crystal layer prevent water molecules from interacting with the surface of the crystal layer, thereby inhibiting its hydration expansion. In addition, the electrostatic interaction of the cations with the surface of the crystal layer of the sample also inhibits the hydration of the cations [35]. In summary, it can be seen that the higher the surface charge density of the cations were, the more obvious the inhibitory effect on the hydration expansion of PS was. This conclusion is similar to that in the literature [36].

### 2.3. Zeta Potential

**Figure 5:**
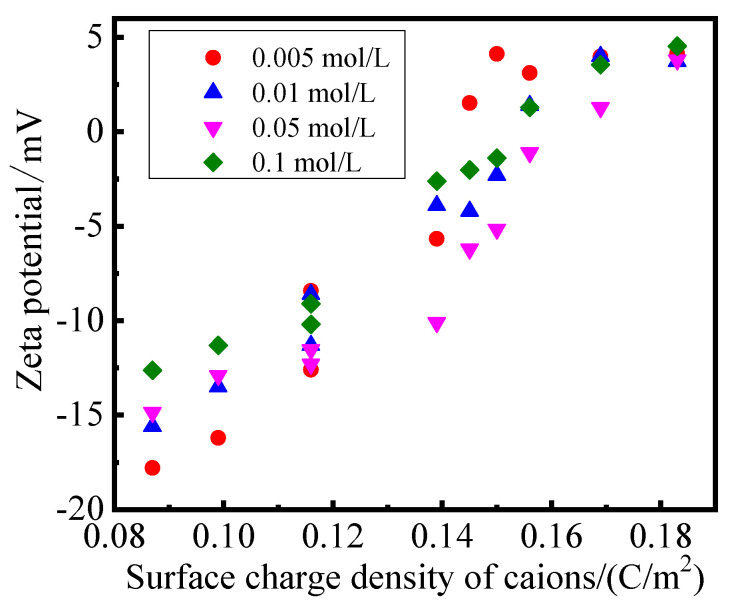
Relationship between surface charge density of cations and zeta potential.

The relationship between the surface charge density of cations and the zeta potential of the modified PS samples at 28 days was presented in Figure 5.

The surface of the PS crystal layer was negative due to the substitution of the crystal lattice, and the zeta potential of PS was −24.5 mV. For the modified PS samples, cations, with a higher surface charge density, would replace ions, with a lower surface charge density, on the surface of the PS crystal layer, and partly neutralize the negative charge on the surface of the crystal layer. The results of Figure 5 showed that the zeta potential of the modified PS samples varied from −18 mV to 5 mV. The zeta potential of the modified PS samples increased with the surface charge density of the cations; this could be explained by the fact that cations with a higher surface charge density have a stronger effect on the surface of the crystal layer and a greater adsorption force [37]. Thus, the more cations adsorbed on the surface of the crystal layer, the stronger the neutralization of the negative charge on the surface of the crystal layer, and the closer the corresponding zeta potential value was to zero. Therefore, the solidification effect and the volume reduction rate of the modified PS samples should enhance with the surface charge density of cations in the modifier solution.

### 2.4. Shear Strength

**Figure 6:**
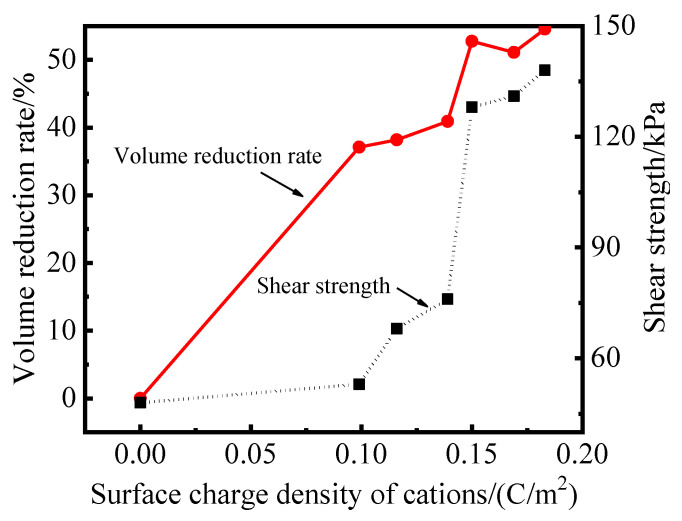
Relationship between the expansion volume, shear strength and surface charge density of cations.

The shear strength of modified PS samples based on NaCl, KCl, MgCl_2_, CaCl_2_, AlCl_3_ and FeCl_3_ at a concentration of 0.05 mol/L at 28 days was shown in Figure 6. The results depicted in Figure 6 showed a similar tendency to that of the zeta potential test. It can be seen that the volume reduction rate and shear strength of the modified PS samples have a good correlation with the surface charge density of cations. With the increase of the surface charge density of cations, the volume reduction rate increased from 0% to 54.55%, and the shear strength increased from 48 kPa to 138 kPa. The results displayed in Figure 6 also show that the hydration expansion of PS was inhibited, the modified PS samples solidified and became denser via ion exchange and led to a significant increase in shear strength.

### 2.5. XRD 

**Figure 7:**
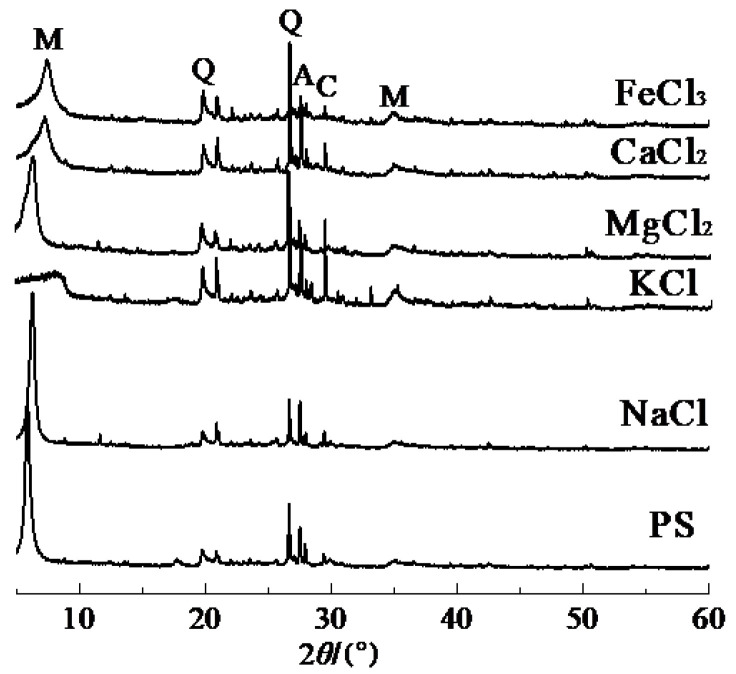
XRD of modified PS samples based on different modifiers. Q, Quartz (SiO_2_); C, Calcite (CaCO_3_); M, Montmorillonite (Ca_0.2_(Al,Mg)Si_4_O_10_(OH)_2_·4H_2_O); A, Albitc (NaAlSi_3_O_8_).

Figure 7 displays the XRD test results of PS and modified PS samples. As shown in Figure 7, the main minerals of PS were quartz (SiO_2_, PDF#01-065-0466), feldspar (NaAlSi_3_O_8_, PDF#01-009-0466), carbonate (CaCO_3_, PDF#01-047-1743) and montmorillonite (Ca_0.2_(Al,Mg)_2_Si_4_O_10_(OH)_2_∙4H_2_O, PDF#01-013-0135). It can be seen that the mineral composition of PS had a negligible change after the modification, but the diffraction peak intensity of montmorillonite had a significant change. The main peak of montmorillonite in PS near *d* = 1.53 nm (6°2*θ*) shifted to a high angle direction between 7°2*θ*~9°2*θ*, and the intensity of the peak decreased and became gentle. It can be seen that the crystal layer spacing has been significantly reduced for the modified PS samples, and the reduction of the crystal layer spacing could effectively inhibit the hydration expansion of samples. It should be noted that, for the sample based on KCl, the intensity of the main broad hump around 6°2*θ* corresponding to montmorillonite decreased sharply and shifted from 6°2*θ* to 9°2*θ*. This decrease and shift of peak could be attributed to the K^+^ that entered the crystal lattice and was well embedded into it due to its ion size close to the size of thecrystal lattice [35,38]. The peaks of quartz (SiO_2_, 26.6°2*θ*) and feldspar (NaAlSi_3_O_8_, 27.9°2*θ*) showed little change from the original materials, and this indicated that the structures of quartz and feldspar had not been altered by the modifiers.

### 2.6. TG/DTG Analysis

**Figure 8:**
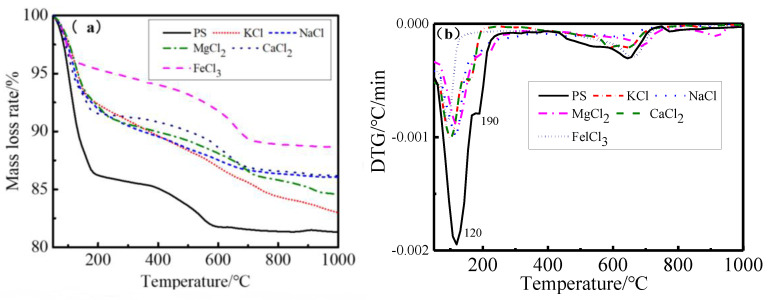
TG/DTG of modified PS samples. (**a**) TG results, (**b**) DTG results.

Figure 8 shows the TG and DTG curves of PS and modified PS samples based on KCl, NaCl, MgCl_2_, CaCl_2_and FeCl_3_. For the PS samples, there were two weight loss stepsin the range of 105–300 °C and 600–800 °C. The total weight loss of the PS samples in the temperature range of 105–300 °C was 8.16%, the weight loss in the range of 105–300 °C for PS samples can be assigned to the drying of the absorbed water and the dihydroxylation of structural water from between the layers, and the weight loss at 600–800 °C was the decomposition of calcite. For modified PS samples based on KCl, NaCl, MgCl_2_, CaCl_2_ and FeCl_3_, the total weight loss values in the range of 105–300 °C were 6.20%, 6.17%, 6.63%, 4.57% and 1.74%, respectively. It can be seen that weight loss significantly decreases at 105–300 °C due to the free water and loosely bound water being sharply reduced after ion exchange modification: weight loss decreased from 8.16% to 6.63%~1.74%. This indicated that the hydration expansion properties of the modified PS samples reduced significantly; on the other hand, the volume stability and shear strength of samples were improved via ion exchange modification, and this was consistent with the results of the sample volume reduction rate and shear strength.

### 2.7. Microstructure Analysis

**Figure 9:**
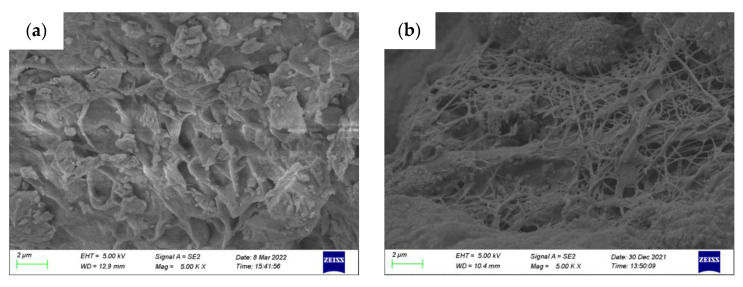
SEM of modified PS samples. (**a**) Sample based on CaCl_2_, (**b**) sample based on AlCl_3_.

Scanning electron microscopy (SEM) was used to analyze the microstructure and hydration product features of the modified PS samples hydrated for 28 days. Figure 9a,b displays the SEM micrographs of the modified PS geopolymer materials based onCaCl_2_ and AlCl_3_, respectively. In Figure 9, many cotton flocs, flakes and flocculent gel substances were observed. It can be seen from Figure 9a that the sample based on CaCl_2_ has a dense structure and shows a limited amount of geopolymer gel; the block crystals and flocculent clay mineral were surrounded by geopolymer gel and mixed together. Figure 9b shows that the reaction produced a homogeneous and filamentous geopolymer gel. The microstructure of the matrix became denser and more homogeneous via ion exchange solidification, and this explains the strength development of the modified PS geopolymers.

## 3. Conclusions

This work reports an investigation of the possibility of developing engineering materials using Pisha sandstone via ion exchange modification. The effect of curing age and modifier types on mechanical properties, volume stability and phase composition were studied. The following conclusions can be drawn:

(1) It is possible to develop engineering materials utilizing Pisha sandstone via ion exchange modification. The study of the properties of modified PS confirmed that the volume stability of samples can be enhanced after the ion exchange modification: the volume of the modified PS sample stabilized after 7 days for the samples based on monovalent modifiers (KCl, NaCl, NH_4_Cl and LiCl), divalent modifiers (CaCl_2_, MgCl_2_, CuCl_2_ and BaCl_2_) and trivalent modifiers (FeCl_3_ and AlCl_3_).The volume reduction rates of samples were 12~39.82%, 14.55~52.73% and 32.55~54.55%, respectively. The volume reduction rates of samples based on monovalent and divalent modifiers reached the maximum value when the modifier solution concentration was 0.05 mol/L, and the maximum value of the volume reduction rate of samples was achieved at the concentration of the trivalent modifiers at 0.01 mol/L.
(2) The types, radius and surface charge density of cations of modifiers had significant effects on the hydration expansion of the samples. The volume reduction rate and mechanical strength of the samples increased with the increase of the surface charge density of cations. The volume reduction rate of samples increased from 5% to 55% when the surface charge density of cations increased from 0.08 C/m^2^ to 0.18 C/m^2^, and the shear strength of the modified PS samples increased from 48 kPa to 138 kPa with the increase of the sample volume reduction rate from 5% to 55%.
(3) The XRD and TG–DTG results of the modified PS samples showed that the diffraction peak of the expansion mineral montmorillonite shifted from 6°2*θ* to 8°2*θ*, and the interlayer spacing was reduced from *d* = 1.53 nm to *d* = 1.07 nm. At the same time, the mass loss in the range of 105~300 °C decreased from 8.16% to 1.74%, the hydration swelling capacity of PS was significantly reduced, and the amount of free water and weakly bound water in the samples was significantly reduced. SEM results showed that the microstructure of the matrix became denser and more homogeneous, and cotton flocs, flakes and flocculent gel substances were the main products.

## 4. Materials and Methods

### 4.1. Materials

**Figure 10:**
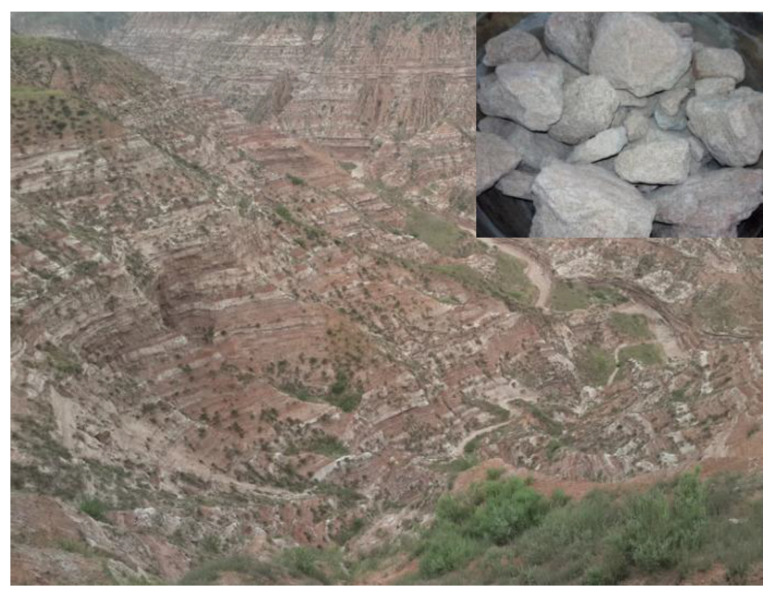
Features of PS area and PS.

**Figure 11:**
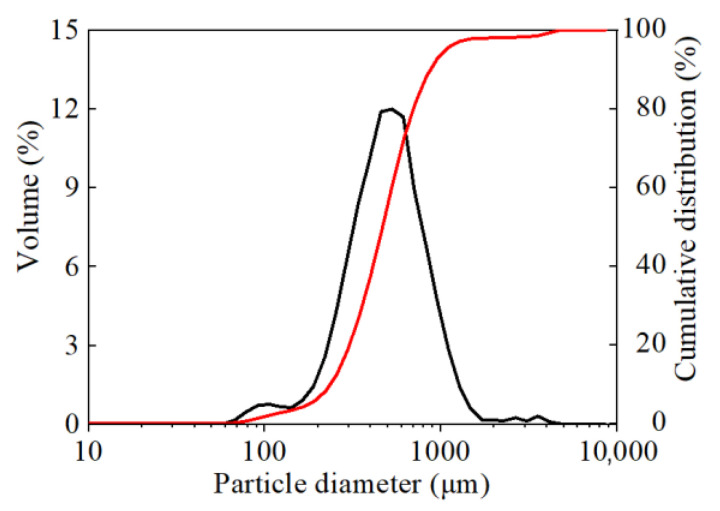
Particle size distribution curve of PS.

In this work, the PS used was obtained from Loess Plateau in Erdos, Inner Mongolia, China. The features of PS area and PS are shown in Figure 10. The particle size distributions of PS shown in Figure 11 were determined by using laser diffraction (Mastersizer 2000, Malvern Instruments, International Joint Research Lab for Eco-building Materials and Engineering of Henan, Henan, China). After drying the wet samples at 105 °C for 24 h, refined PS was obtained by pulverization. The mineral and chemical compositions of PS were determined by X-ray diffraction (XRD) and X-ray fluorescence (XRF) techniques (Bruker, Guangzhou, China), respectively. The detailed chemical compositions are shown in Table 1. The modifier solutions were prepared by dissolving KCl, NaCl, NH_4_Cl, LiCl, MgCl_2,_CaCl_2_, CuCl_2_, BaCl_2_, AlCl_3_ and FeCl_3_ pellets (99% purity quotient, Tianjin Kemiou Chemical Reagent Co., Ltd., Tianjin, China) in distilled water to a certain concentration (Table 2).

### 4.2. Sample Preparation and Characterization

After adding 2.0 g PS powder into a measuring cylinder (precision is 0.2 mL) with cover containing 45mL modifier solution (e.g., KCl, NaCl, NH_4_Cl, LiCl, MgCl_2,_ CaCl_2_, CuCl_2_, BaCl_2_, AlCl_3_ and FeCl_3_), the prepared samples were cured at ambient temperature (25 ± 2 °C) and the expansion volume of samples was recorded on days 0.5, 1, 2, 3, 7, 14 and 28. The materials used to prepare the modified PS geopolymer samples were summarized in Table 3. For all samples, the total mass of the mixture was kept at 392.5 g. The PS powder was added into modifier solution (e.g., NaCl, CaCl_2_ and FeCl_3_), in order to achieve the complete mixing between the solid and solution. The mixture was mixed for 15 min with amagnetic stir bar. To produce regularly shaped samples for mechanical testing, the mixtures were poured into cylindrical steel molds and pressed to samples with a bar of a diameter of 5 cm and height of 10 cm (i.e., an aspect ratio of 2.0) on a hydraulic testing machine. To ensure repeatability, three samples were prepared for each type of modified PS geopolymer sample. The samples were cured at ambient temperature for 28 days.

A ZetaPlus Zeta Potential Analyzer (ZETAPLUS) was used to measure the zeta potential value of modified PS powder which had been immersed in modifier solution for 28 days. The Zeta Plus system measured electrophoretic mobility by light scattering. The shearing strength of modified PS geopolymer samples cured for 28 days was measured in triplicate by direct shear tests (Table 3). The shear strength of samples hydrated was measured by a controls multipurpose electronic universal testing machine with a load cell of 100 kN capacity. The vertical pressures for the direct shear tests were 100, 200, 300 and 400 kPa, respectively. In the direct shearing, a velocity of 0.005 mm/min in the horizontal direction was chosen during shearing. The compositions of modified PS powders were evaluated by XRD, and the XRD was recorded on a Siemens/Bruker D5000 using Cu *K*α radiation (λ = 1.54 Å) operating at 40 kV and 30 mA. The samples were scanned from 5 to 60° (2*θ* range) at a rate of 2°/min and step size of 0.02°. A Mettler Toledo (Switzerland) simultaneous thermal analyzer was used to measure some of the physical properties of modified PS powders as a function of the temperature change. For thermogravimetric analysis, the samples were heated in an atmosphere of nitrogen at 10 °C min^−1^ from 40 to 1000 °C.

## Figures and Tables

**Table 1:** Composition of PS (XRF).

SiO_2_	Al_2_O_3_	Fe_2_O_3_	TiO_2_	CaO	MgO	K_2_O	Na_2_O	MnO	FeO	CO_2_	LOI
64.7	10.1	1.8	0.3	7.4	1.2	2.7	1.4	0.1	1.8	5.40	3.1

**Table 2:** Parameter of modifier solution.

Modifier Type	Concentration/(mol/L)			
KCl	0.005	0.01	0.05	0.1
NaCl	0.005	0.01	0.05	0.1
NH_4_Cl	0.005	0.01	0.05	0.1
LiCl	0.005	0.01	0.05	0.1
MgCl_2_	0.005	0.01	0.05	0.1
CaCl_2_	0.005	0.01	0.05	0.1
CuCl_2_	0.005	0.01	0.05	0.1
BaCl_2_	0.005	0.01	0.05	0.1
AlCl_3_	0.005	0.01	0.05	0.1
FeCl_3_	0.005	0.01	0.05	0.1

**Table 3:** Parameter of modified PS geopolymer samples.

Number	Modifier Type	Modifier Solution		Solid Amount/(%) *s*/*b* ^#^	
		Concentration/Amount (mol/L)/(%)			
1	Water	0	20	80	0.25
2	NaCl	0.05	20	80	0.25
3	CaCl_2_	0.05	20	80	0.25
4	FeCl_3_	0.05	20	80	0.25

^#^ The weight ratio of modifier solution (including modifier pellets and water) and solid mixture (PS).

## Data Availability

Data obtained as described.
